# Tuning Photophysical Properties via Positional Isomerization of the Pyridine Ring in Donor–Acceptor-Structured Aggregation-Induced Emission Luminogens Based on Phenylmethylene Pyridineacetonitrile Derivatives

**DOI:** 10.3390/molecules28073282

**Published:** 2023-04-06

**Authors:** Haiya Sun, Shuixin Chen, Aiguo Zhong, Rong Sun, Jiajie Jin, Jiahao Yang, Dongzhi Liu, Junfeng Niu, Shengli Lu

**Affiliations:** 1 Key Laboratory of Chemical and Biological Processing Technology for Farm Products of Zhejiang Province, School of Biological and Chemical Engineering, Zhejiang University of Science and Technology, Hangzhou 310023, China; chenshuixin02@163.com (S.C.); 115069@zust.edu.cn (R.S.); 1200450057@zust.edu.cn (J.J.); yjh19858872471@163.com (J.Y.); 103044@zust.edu.cn (J.N.); 2 School of Pharmaceutical and Chemical Engineering, Taizhou University, Taizhou 318000, China; zhongaiguo@tzc.edu.cn; 3 School of Chemical Engineering and Technology, Tianjin University, Tianjin 300072, China; dzliu@tju.edu.cn

**Keywords:** aggregation-induced emission, phenylmethylene pyridineacetonitrile, positional isomerization, fluorescence emission, non-doped organic light-emitting diode

## Abstract

A series of aggregation-induced emission (AIE)-featured phenylmethylene pyridineacetonitrile derivatives named ***o*-DBCNPy** ((*Z*)-3-(4-(di-*p*-tolylamino)phenyl)-2-(pyridin-2-yl)acrylonitrile), ***m*-DBCNPy** ((*Z*)-3-(4-(di-*p*-tolylamino)phenyl)-2-(pyridin-3-yl)acrylonitrile), and ***p*****-DBCNPy** ((*Z*)-3-(4-(di-*p*-tolylamino)phenyl)-2-(pyridin-4-yl)acrylonitrile) have been synthesized by tuning the substitution position of the pyridine ring. The linkage manner of the pyridine ring had influences on the molecular configuration and conjugation, thus leading to different photophysical properties. The absorption and fluorescence emission peak showed a bathochromic shift when the linking position of the pyridine ring changed from the *meta* to the *ortho* and *para* position. Meanwhile, ***o*-DBCNPy** exhibited the highest fluorescence quantum yield of 0.81 and the longest fluorescence lifetime of 7.96 ns as a neat film among all three isomers. Moreover, non-doped organic light-emitting diodes (OLEDs) were assembled in which the molecules acted as the light-emitting layer. Due to the relatively prominent emission properties, the electroluminescence (EL) performance of the ***o*-DBCNPy**-based OLED was superior to those of the devices based on the other two isomers with an external quantum efficiency (EQE) of 4.31%. The results indicate that delicate molecular modulation of AIE molecules could endow them with improved photophysical properties, making them potential candidates for organic photoelectronic devices.

## 1. Introduction

In recent years, the solid-state luminescence of organic materials has drawn much attention due to the rapid development of optoelectronic devices and applications such as organic light emitting diodes (OLEDs) [1,2,3], optical sensors [4], stimuli responses, and anti-counterfeit printing [5]. However, many organic materials exhibit intense fluorescence emission in dilute solutions, while the emission is severely quenched in the aggregate state. This undesirable aggregation-caused quenching (ACQ) effect has, to a large extent, restricted the applications of organic fluorescence materials in the solid state [6,7]. Fortunately, molecules with aggregation-induced emission (AIE) characteristics, which were first reported in 2001 by Tang’s group, have shown their superiority in terms of strong fluorescent emission and high photostability in the aggregate state [8,9,10]. The concept of AIE describes a unique phenomenon in that some organic fluorophores are weakly or non-emissive in a molecularly dissolved state (dilute solution), but intensely emissive in aggregate (solid) states [11]. The mechanism responsible for the AIE phenomenon is currently considered to be the restriction of intramolecular motions (RIM) [12,13,14]. As such, AIE luminogens (AIEgens) are usually characterized by propeller-shaped peripheral intramolecular rotors [15] or twisted configurations [16].

To date, several AIEgens, including silole [17], tetraphenylethene (TPE) [18], distyrylanthracene [19], tetraphenylpyrazine [20], quinoline-malononitrile [21], and cyanostilbene [22], have been reported. To exploit the fluorescence properties of AIEgens in aggregate states and to promote their application in solid-supported materials and devices, several strategies have been proposed in terms of molecular design. One strategy is to develop AIEgens with novel structures additional to the “traditional” silole and TPE core. For instance, chiral AIEgens based on 1,1′-bi-2-naphthol, metal complexes, and other chiral alkyl chains were reported and applied in circularly polarized organic light-emitting diodes (CPOLEDs) to pursue higher contrast and better emission efficiency in 3D displays [23]. Diarylethenes containing a benzobis(thiadiazole) linkage with large steric hindrance have been reported, of which some compounds show excellent potential for super-resolution imaging [24]. A series of organometallic or coordination AIEgens have been synthesized as mechanochromic luminescence (MCL) materials with better photophysical properties, richer emission colors, and more controllable MCL effects [25]. AIE polymers characterized by fused heterocyclic building blocks were also developed for the practical application of morphological structure visualization (e.g., microphase separation of polymer blends) and external stimuli response [26]. These research efforts have paved the way for the development of AIE-based optoelectronic devices, yet the synthetic complexity and material cost (such as the molecules containing noble metals) are currently the limitations for the broader applications of these AIEgens. Another strategy is the systematic structural tuning of the present AIEgens to optimize the solid-state luminescent properties. For organic fluorophores that are facilely modified, the substitution position effect of the functional groups or building blocks significantly affects the conjugation, intramolecular charge distribution, and transfer, and subsequently the photophysical properties [27,28,29,30,31,32,33,34,35,36,37]. Though some research has been performed that has provided the optimized substitution manner of the AIEgens, which led to the enhanced performance of the solid photoelectronic devices [38,39,40], more detailed investigations are still in demand to reveal and to further manipulate the detailed structure¬–property relationship.

To this end, a series of phenylmethylene pyridineacetonitrile derivatives bearing a triphenylamine (TPA) structure and pyridine ring with the nitrogen atom at the *ortho* (***o*-DBCNPy**), *meta* (***m*-DBCNPy**), and *para* (***p*-DBCNPy**) position were designed and synthesized. The propeller-like TPA moiety was introduced as a strong electron donor and to prevent intimate π–π intermolecular stacking. The acceptor strength was tuned by varying the linking manner of the pyridyl ring. The photophysical properties of the compounds in solutions and the solid state were investigated. The emission performances in solid-state applications, including mechanofluorochromism and non-doped OLEDs, were studied and correlated to the substitutional position of the pyridyl group.

## 2. Results and Discussions

### 2.1. Synthesis and Characterization

**Scheme 1:**
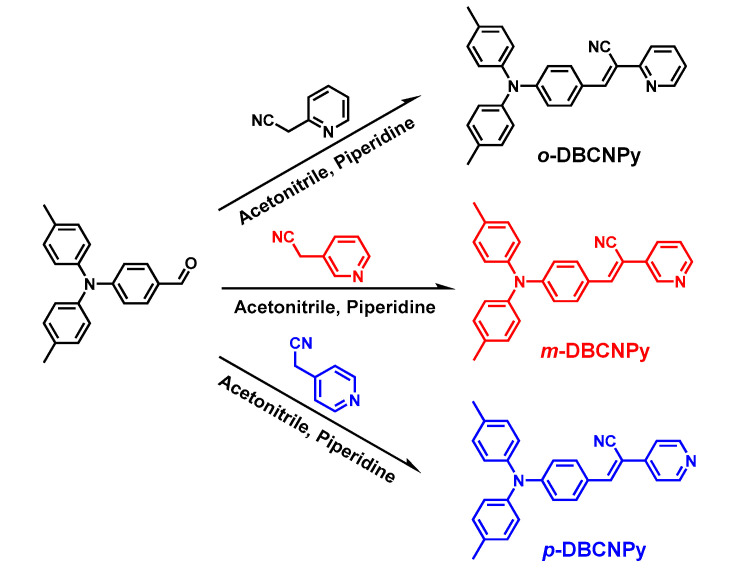
Synthetic routes of the compounds.

The molecules were synthesized through Knoevenagel condensation between pyridinyl acetonitriles and 4-(di-*p*-tolylamino)benzaldehyde with yields of 65–75% (Scheme 1), and their structures were characterized by ^1^H NMR, ^13^C NMR, and HRMS (Figures S1–S6, Supplementary Materials). For solid fluorescence materials, thermal stability was a prerequisite before they were employed in the devices. Thermogravimetric analysis (TGA) results of the compounds are shown in Figure S7. The thermal decomposition temperatures (*T*_d_, 5% weight loss) for ***o*-DBCNPy**, ***m*-DBCNPy**, and ***p*-DBCNPy** were 375 °C, 344 °C, and 343 °C, respectively, showing relatively good thermal stability.

### 2.2. Spectral Analysis

**Figure 1:**
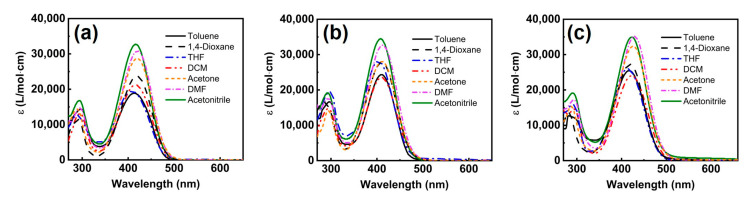
Absorption spectra of ***o*-DBCNPy** (**a**), ***m*-DBCNPy** (**b**), and ***p*-DBCNPy** (**c**) in 1 × 10^−5^ M solutions (THF, tetrahydrofuran; DCM, dichloromethane; DMF, *N*, *N*-dimethylformamide).

Figure 1 shows the UV-vis absorption spectra of the compounds in solvents with different polarities. Two absorption peaks can be observed for all three molecules at around 295 nm and in the range of 400–420 nm: the former was ascribed to the TPA-localized electronic π–π* transition [41,42], and the latter with a longer wavelength was due to the intramolecular charge transfer (ICT) resulting from electronic transition [43,44]. The absorption peak wavelength was barely shifted in the different solvents, indicating that the ground state charge transfer was hardly affected by the solvent polarity. Among the three molecules, ***p*-DBCNPy** showed the most redshifted absorption peak, which was due to the increased conjugation between the *para*-pyridine and the TPA-acrylonitrile part [30]. Specifically, ***p*-DBCNPy** had the largest absorption peak wavelength in THF (413 nm) in comparison to those of ***o*-DBCNPy** (408 nm) and ***m*-DBCNPy** (402 nm).

**Figure 2:**
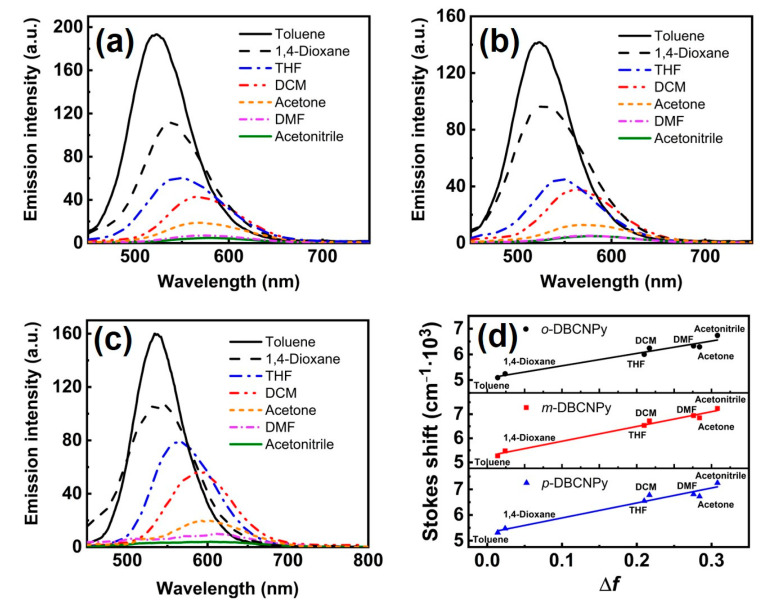
Fluorescence emission spectra of ***o*-DBCNPy** (**a**), ***m*-DBCNPy** (**b**), and ***p*-DBCNPy** (**c**) in 1 × 10^−5^ M solutions (THF, tetrahydrofuran; DCM, dichloromethane; DMF, *N*, *N*-dimethylformamide); the plot of Stokes shifts of the three isomers in different solvents versus solvent orientation polarizability (Δ*f*) (**d**).

The fluorescence emission spectra of the molecules are shown in Figure 2a–c. The solvent polarity had a large effect on the emission. On the one hand, a general trend was observed for all three fluorophores that the fluorescence emission peak was redshifted (62 nm for ***o*-DBCNPy**, 57 nm for ***m*-DBCNPy**, and 81 nm for ***p*-DBCNPy**, respectively) and in the meantime, the emission intensity was diminished when the solvent polarity increased (from toluene to acetonitrile). The trend of increasing Stokes shift with the enhancement of the solvent polarity was also illustrated by the Lippert–Mataga model. As shown in Figure 2d, Stokes shifts of the molecules in different solvents rose linearly with a slope of over 5000 as the solvent orientation polarizability (Δ*f*) grew, indicative of an obvious solvatochromic effect. On the other hand, the substituent positions of the pyridine ring also affected the emission property. For ***p*-DBCNPy**, the best conjugation and the strongest donor–acceptor interaction result in the most redshifted emission peak (566 nm in THF). In comparison, due to the less conjugated *meta*-linkage mode, the fluorescent emission peak wavelength was 25 nm shorter for ***m*-DBCNPy** in THF. The photophysical data of the isomers in THF are summarized in Table 1.

**Figure 3:**
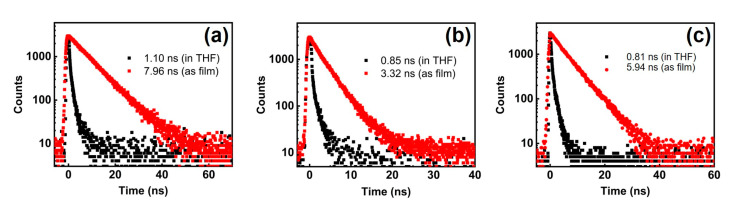
Fluorescence lifetimes of ***o*-DBCNPy** (**a**), ***m*-DBCNPy** (**b**), and ***p*-DBCNPy** (**c**) in THF and as neat film.

The photophysical properties of the molecules at solid state (as neat film) were investigated. Figure S8 shows that the absorption bands of the compound neat films were around 410 nm, and the emission peak wavelengths were slightly different (543 nm for ***o*-DBCNPy**, 541 nm for ***m*-DBCNPy**, and 548 nm for ***p*-DBCNPy**). The fluorescence lifetimes of the solid-state compounds were elongated in comparison to those in solutions (Figure 3). Specifically, the ***o*-DBCNPy** film showed the longest fluorescence lifetime of 7.96 ns, 7.2 times extended when compared to the emission lifetime in THF. Meanwhile, the fluorescence quantum yields of the neat films of all compounds (*Φ*_f,f_) were measured. ***O*-DBCNPy** film was highly emissive, with the *Φ*_f,f_ reaching 0.81, showing good potential as an organic light-emitting material. In comparison, ***m*-DBCNPy** showed the lowest *Φ*_f,f_ (0.49) among all three isomers, which was attributed to its short fluorescence lifetime. Overall, the enhanced emission intensity and the extended fluorescence lifetime of the non-doped films indicates that the formation of the aggregate state may affect the decay pathway of the excited state, i.e., suppressing the nonradiative decay [45,46,47]. The different fluorescence lifetimes and quantum yields among the isomers were probably derived from the varied molecular configurations and conjugations in the solid state due to the substitutional position of the pyridine ring [48]. For ***o*-DBCNPy**, the strongest emission intensity might be derived from a more planarized molecular configuration led by the interaction between the *ortho*-nitrogen atom and the vinyl hydrogen atom (-CH=C-). The fluorescence emission data of the compound films are listed in Table 2.

### 2.3. Aggregation-Induced Emission (AIE)

**Figure 4:**
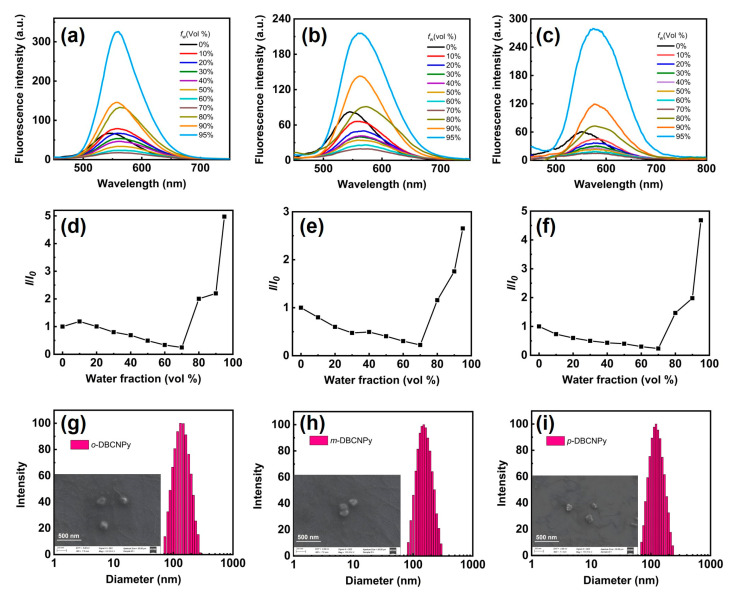
Fluorescence spectra, variation in relative emission intensity (*I*/*I*_0_), and emission maxima wavelength of ***o*-DBCNPy** (**a**,**d**), ***m*-DBCNPy** (**b**,**e**), and ***p*-DBCNPy** (**c**,**f**) in THF–water mixtures with increasing *f*_w_ (concentration of compound: 1 × 10^−5^ M, *λ*_ex_ = 415 nm); size distributions of ***o*-DBCNPy** (**g**), ***m*-DBCNPy** (**h**), and ***p*-DBCNPy** (**i**) aggregates in THF–water mixtures (*f*_w_ = 95%) (insets are SEM images of the particles).

To understand the spectral properties of the compounds from solutions to aggregate state, the absorption and fluorescence emission spectra were recorded in the THF (good solvent)–water (poor solvent) mixtures with increasing water fractions (*f*_w_, by volume %). As shown in Figure S9, the absorption of the compounds was barely affected when *f*_w_ was below 70%. When the water ratio further increased, the absorption band was redshifted with a level-off absorption tail extended to a longer wavelength. This was due to the Mie scattering by the nano-aggregates of the molecules at high *f*_w_ [49]. The fluorescence emission spectra of all isomers are shown in Figure 4a–c, and the variations in emission intensity and peak position with respect to the *f*_w_ are also shown in Figure 4d–f. As the *f*_w_ increased to 70–80%, the emission intensity steadily dropped with the fluorescence quantum yield in solution (*Φ*_f,s_), decreased to less than 0.02, and the emission maximum wavelength shifted bathochromatically. The diminished fluorescence emission can be explained by the twisted intramolecular charge transfer process of a typical donor–acceptor (D–A) fluorophore in solvents with high polarity [50]. The redshifted emission was due to solvatochromism. When *f*_w_ increased, the fluorescence emission was intensified drastically. At *f*_w_ = 95%, the dispersions of the three molecules showed significantly increased *Φ*_f,s_ values of 0.25–0.34. This could be attributed to the formation of aggregates [47,51]. Considering that (1) the molecules were highly emissive in the solid state and (2) they showed significantly enhanced fluorescence emission during the aggregate formation, the DBCNPy isomers belonged to typical AIE molecules.

Moreover, the formation of the nano-aggregates of the isomers in THF–water mixtures at high *f*_w_ is evidenced by the results from the dynamic light scattering (DLS) and scanning electron microscopy (SEM) characterizations. Figure 4g–i shows that the average sizes of the aggregates were within the range of 120–150 nm. The particles can also be observed in the inset SEM images, which support the DLS data.

### 2.4. Electronic Properties

**Figure 5:**
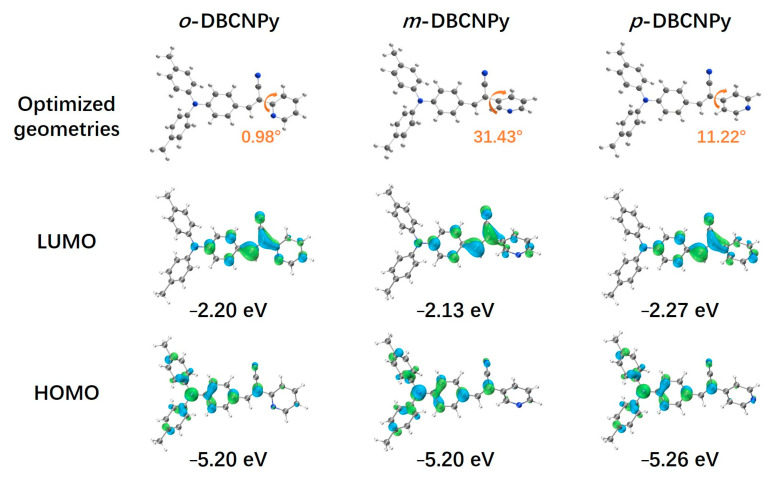
Optimized molecular geometries, electron density distributions, and energy levels of the HOMO and LUMO orbitals for the molecules at the B3LYP/6-311g(d) basis set.

To gain insight into the molecular structures and the electronic properties of the DBCNPy isomers, density functional theory (DFT) calculations were performed with the Gaussian 09 program at the B3LYP/6-311g(d) basis set [52]. The optimized molecular geometries (Figure 5) revealed the different planarities of the isomers’ acceptor moieties. The torsion angle between the pyrimidine ring and the central -C=C-plane increased from 11.22° in ***p*-DBCNPy** to 31.43° in ***m*-DBCNPy**, which was presumably due to the repulsion between the cyano group and the lone-pair electrons on the *meta*-nitrogen atom. In ***o*-DBCNPy**, however, the torsion angle was decreased to only 0.98°. This could be attributed to the interaction between the *ortho*-nitrogen atom and the hydrogen on the vinyl group (-CH=C-) The torsion extent of the pyridine ring in the three isomers might partly explain the differences in their fluorescence properties: higher planarity between the imide and the pyridine led to elongated fluorescence lifetime and more intense fluorescence emission. In addition, the distributions of the frontier molecular orbitals are also depicted in Figure 5. The highest occupied molecular orbitals (HOMOs) and lowest unoccupied molecular orbitals (LUMOs) of the isomers were spatially separated, with the former mainly located on the TPA moiety and the latter distributed on the imide and pyridine part, demonstrating the D–A feature of the isomers [53].

**Figure 6:**
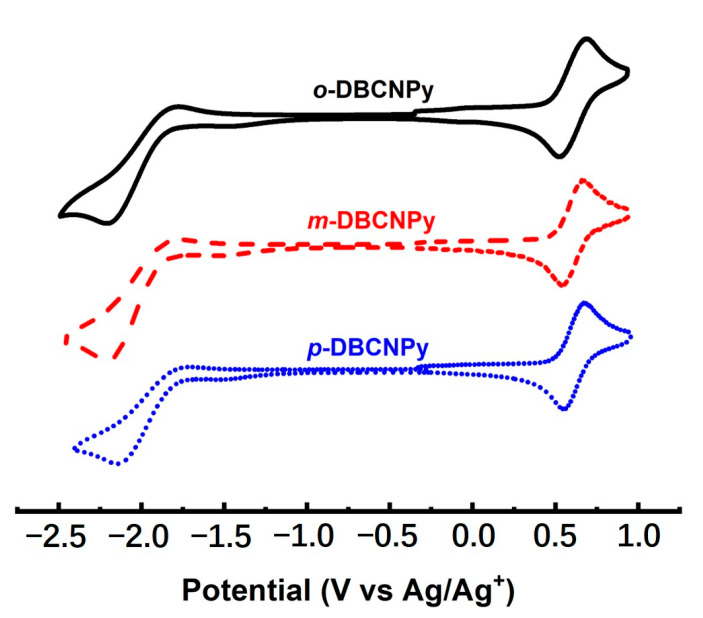
Cyclic voltammograms of the compounds; scan rate, 50 mV s^−1^.

Cyclic voltammetry (CV) analysis was carried out and the CV curves of the fluorophores were recorded (Figure 6). The oxidation potential (*E*_ox_) of the three molecules from the quasi-reversible oxidation peak of the triphenylamine part were similar (0.60–0.62 V). The reduction potentials (*E*_red_) showed a slight difference among the isomers, and were determined to be −1.98V, −2.01V, and −1.93V for ***o*-DBCNPy**, ***m*-DBCNPy**, and ***p*-DBCNPy**, respectively. Accordingly, the vertical ionization potential (IP) and the vertical electron affinity (EA) were estimated by the equations IP = 4.93 eV + *E*_ox_ and EA = 4.93 eV + *E*_red_ [54,55], and the data are given in Table 3. The results indicate that the *para*-pyridine showed a stronger electron-accepting ability than the other two isomers. This is also in accordance with the results of the spectral analysis. Notably, the IP and EA values matched well with most of the hole- and electron-transporting materials, meaning that these isomers were energetically appropriate to be assembled into electroluminescence devices as emitting materials.

### 2.5. Mechanofluorochromism

**Figure 7:**
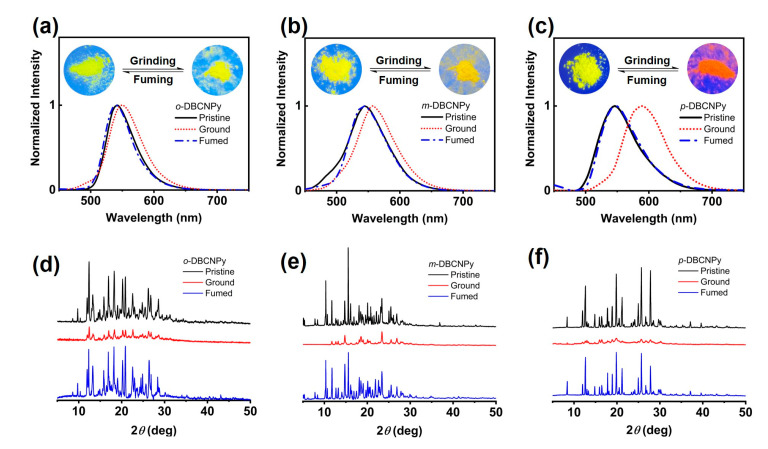
Fluorescence emission spectra and XRD patterns of ***o*-DBCNPy** (**a**,**d**), ***m*-DBCNPy** (**b**,**e**), and ***p*-DBCNPy** (**c**,**f**) when solid at various states (pristine, ground, and fumed); insets are images of the each solid under 365 nm UV light.

Considering that ***p*-DBCNPy** was relatively sensitive toward the stimulus of external force, the mechanofluorochromic (MFC) properties of the other two isomers were also investigated, and their emission spectra and powder X-ray diffraction (XRD) patterns at different states are demonstrated in Figure 7. Unfortunately, the MFC effect of ***o*-DBCNPy** and ***m*-DBCNPy** was not as prominent as that of ***p*-DBCNPy**. Although ***o*-DBCNPy** and ***m*-DBCNPy** showed good reversibility during grinding and solvent fuming, the wavelength changes were no more than 20 nm. This was probably due to a smaller change in the powder morphology under external force: partially losing their crystallinity instead of fully converting into an amorphous form [56]. After hard grinding, the XRD patterns of ***o*-DBCNPy** and ***m*-DBCNPy** retained some features of a microcrystalline structure, as shown in Figure 7d,e. Furthermore, the differential scanning calorimetry (DSC) curves (Figure S10) show only weak exothermic peaks at 93.5 °C for ***o*-DBCNPy** and 99.7 °C for ***m*-DBCNPy**, indicative of a smaller extent of the cold crystallization process than that of ***p*-DBCNPy [57]**. Meanwhile, although attempts were made to grow single crystals of ***o*-DBCNPy** and ***m*-DBCNPy**, the needle-like crispy microcrystals were unable to be analyzed by single-crystal X-ray diffraction. In comparison, ***p*-DBCNPy** easily formed well-ordered microcrystals or even single crystals through slow solvent evaporation, and the orderly arranged molecules transformed into an amorphous state upon grinding (Figure 7f). The results indicate that the substitution position of the pyridine ring could affect the crystallinity and MFC property of the molecule series.

### 2.6. OLED Device Performances

**Figure 8:**
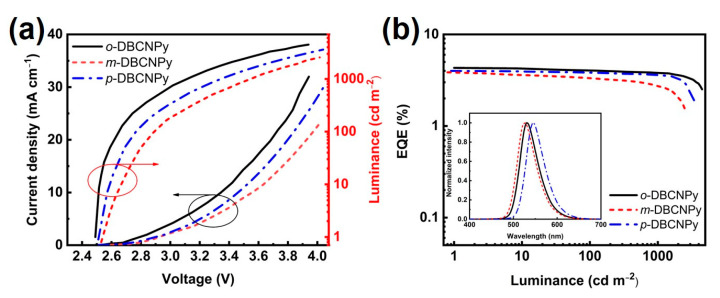
Current density–voltage–luminance (*J*–*V*–*L*) (**a**) and EQE-luminance characteristics (**b**) for the devices (inset showed the EL spectra).

Non-doped organic light-emitting diode (OLED) devices based on the three molecules were fabricated due to their relatively high fluorescence quantum yields in solid state (film) and appropriate IPs and EAs. The devices were assembled based on the following configuration: ITO/MoO_3_ (hole injection layer, 3 nm)/TAPC (hole-transporting layer, 50 nm)/TCTA (exciton-blocking layer, 10 nm)/DBCNPy isomers (light-emitting layer, 25 nm)/TPBi (electron-transporting layer, 50 nm)/LiF (electron injection layer, 1 nm)/Al [58]. The electroluminescence (EL) characteristics of the devices are demonstrated in Figure 8, and the data are summarized in Table 4. All OLEDs emit green light with a brightness exceeding 2000 cd m^2^, and the light colors of the ***o*-DBCNPy**- and ***m*-DBCNPy**-based devices were close to pure green (color coordinates (0.21, 0.71)). The best electroluminescence performance was achieved by the device base on ***o*-DBCNPy** with a maximum current efficiency (CE), power efficiency (PE), and external quantum efficiency (EQE) of 18.95 cd A^−1^, 15.76 lm W^−1^, and 4.31%, respectively, and the color coordinates are (0.26, 0.70). By comparing the EL performances among the three devices, it was observed that the parameters (especially EQE) were roughly in accordance with the fluorescence quantum yields of the non-doped films (*Φ*_f,f_). In terms of EQE, the ***o*-DBCNPy**-based device was among the best of the traditional fluorescent OLEDs based on cyanostilbene derivatives reported so far [59,60], including doped and non-doped devices. In addition, the EL performances and the structural modification feasibility made it possible to produce white OLEDs using the DBCNPy molecule series as the emitting materials.

## 3. Materials and Methods

### 3.1. Materials and Characterization

Starting materials including the donor molecule 4-(di-*p*-tolylamino)benzaldehyde, the acceptor building block 2-(pyridin-2-yl)acetonitrile, 3-(pyridin-2-yl)acetonitrile, 4-(pyridin-2-yl)acetonitrile, and MoO_3_, LiF, 4,4′-cyclohexylidene bis[*N*, *N*-bis(*p*-tolyl)aniline] (TAPC), 1,3,5-tris(1-phenyl-1*H*-benzimidazol-2-yl)benzene (TPBi), and 4,4′,4″-tris(carbazol-9-yl)-triphenylamine (TCTA) for device fabrication were obtained from Energy Chemical (Shanghai, China), Bide Pharmatech Co., Ltd. (Shanghai, China) and Aladdin Biochemical Technology Co., Ltd. (Shanghai, China), and were used without further treatment. All solvents were of AR grade and were purified by the established procedures.

Nuclear magnetic resonance measurements, including ^1^H NMR (400 MHz) and ^13^C NMR (100 MHz), were performed on Bruker spectrometers at 25 °C. High-resolution mass spectroscopy (HRMS) analysis was performed on a Bruker micrOTOF-Q II mass spectrometer. For spectral analysis, the absorption and fluorescence emission spectra were acquired on a Bruker UV/vis spectrometer and a Bruker fluorescence spectrophotometer, respectively. Time-resolved fluorescence analysis and fluorescence quantum yield measurements were carried out on an Edinburgh FLS1000 (Livingston, UK) fluorescence spectrophotometer. For nano-aggregates, the particle size was measured through dynamic light scattering (DLS) measurements via a Malvern Zetasizer Nano ZS90 (Malvern Panalytical, Malvern, UK) size analyzer and observed using a Zeiss Sigma 300 (Jena, Germany) scanning electron microscope (SEM). The microcrystalline structure of the compounds was analyzed via an X-ray diffraction (XRD) SmartLab (Rigaku, Tokyo, Japan) X-ray diffractometer at room temperature.

### 3.2. Synthesis

General synthetic method of the molecules. The target compounds were synthesized according to a previous report [57]. Briefly, 4-(di-*p*-tolylamino)benzaldehyde (1 equiv.) and (pyridin-2-yl)acetonitrile derivatives (1.2 equiv.) were dissolved in 20–30 mL acetonitrile, and a catalytic amount of piperidine (usually three drops per mmol of the reactant) was added into the solution. The reactant mixture was stirred at 85 °C for 8–10 h and was then filtered. The precipitate was rinsed with cold ethanol and recrystallized in a mixed solvent of cyclohexane and dichloromethane (10:1–15:1, *v*/*v*).

Synthesis and characterization of (*Z*)-3-(4-(di-*p*-tolylamino)phenyl)-2-(pyridin-2-yl)acrylonitrile (***o*-DBCNPy**). ***o*-DBCNPy** was obtained as a bright yellow solid from the starting material 4-(di-*p*-tolylamino)benzaldehyde and 2-(pyridin-2-yl)acetonitrile (yield: 71%). ^1^H NMR (400 MHz, CDCl_3_, δ): 8.60 (d, *J* = 4.4 Hz, 1H), 8.34 (s, 1H), 7.85 (d, *J* = 8.8 Hz, 2H), 7.77–7.69 (m, 2H), 7.23–7.20 (m, 1H), 7.13 (d, *J* = 8.4 Hz, 4H), 7.07 (d, *J* = 8.4 Hz, 4H), 6.97 (d, *J* = 8.8 Hz, 2H), 2.34 (s, 6H). ^13^C NMR (100 MHz, CDCl_3_, δ): 152.02, 150.96, 149.46, 144.62, 143.82, 137.24, 134.53, 131.61, 130.26, 126.10, 125.00, 122.62, 120.76, 119.23, 118.88, 105.18, 20.96. HRMS (ESI) *m/z*: [M + H]^+^ calcd for C_28_H_23_N_3_, 402.1965; found, 402.1961.

Synthesis and characterization of (*Z*)-3-(4-(di-*p*-tolylamino)phenyl)-2-(pyridin-3-yl)acrylonitrile (***m*-DBCNPy**). ***m*-DBCNPy** was acquired as a pale yellow solid from 4-(di-*p*-tolylamino)benzaldehyde and 3-(pyridin-2-yl)acetonitrile (yield: 66%). ^1^H NMR (400 MHz, CDCl_3_, δ): 8.88 (d, *J* = 2.0 Hz, 1H), 8.57 (d, *J* = 4.8 Hz, 1H), 7.92 (d, *J* = 8.0 Hz, 1H), 7.76 (d, *J* = 8.8 Hz, 2H), 7.42 (s, 1H), 7.36–7.30 (m, 1H), 7.14 (d, *J* = 8.4 Hz, 4H), 7.07 (d, *J* = 8.4 Hz, 4H), 6.98 (d, *J* = 8.8 Hz, 2H), 2.35 (s, 6H). ^13^C NMR (100 MHz, CDCl_3_, δ): 150.91, 149.22, 146.78, 143.77, 143.28, 134.59, 133.00, 131.38, 130.98, 130.28, 126.06, 124.86, 123.54, 119.31, 118.22, 103.19, 20.96. HRMS (ESI) *m/z*: [M + H]^+^ calcd for C_28_H_23_N_3_, 402.1965; found, 402.1959.

Synthesis and characterization of (*Z*)-3-(4-(di-*p*-tolylamino)phenyl)-2-(pyridin-4-yl)acrylonitrile (***p*-DBCNPy**). ***p*-DBCNPy** was obtained as a bright yellow crystal from 4-(di-*p*-tolylamino)benzaldehyde and 4-(pyridin-2-yl)acetonitrile (yield: 75%). The characterization data have been reported by [57].

### 3.3. Electrochemical Measurement

The CHI 660E Electrochemical Workstation was used to conduct the measurements of the electrochemical properties. A three-electrode set up, calibrated by utilizing the ferrocene/ferrocenium (Fc/Fc^+^) redox couple, was used in the measurements. The Ag/AgNO_3_ electrode was chosen as the reference electrode. Tetra-butyl ammonium hexafluorophosphate (TBAPF_6_) was dissolved in dichloromethane (0.1 M) as the electrolyte.

### 3.4. Fabrication and Characterization of Electroluminescent Devices

Indium tin oxide (ITO) conducting glass was washed, cleaned ultrasonically, and treated with ultraviolet and ozone prior to use. The compounds were sublimated at the condition of 200 °C and 1 × 10^−3^ Pa. The light-emitting layer, electron/hole transport layer, and electrode were deposited onto the ITO substrates at a pressure of 5 × 10^−4^ Pa. The electroluminescence characteristics and the emission spectra of the devices were measured by a Keithey 2400 (Tektronix, Inc., Beaverton, OR, USA) source meter and a PR655 (North Syracuse, NY, USA) SpectraScan^®^ spectrometer.

## 4. Conclusions

In conclusion, three phenylmethylene pyridineacetonitrile derivatives (***o*-DBCNPy**, ***m*-DBCNPy**, and ***p*****-DBCNPy**) with various substitution positions of the pyridine ring have been synthesized via Knoevenagel condensation in good yields. All three compounds were characterized with aggregation-induced emission (AIE) features since they were highly emissive in the solid state and their fluorescence emission showed significant enhancement during aggregate formation. The positional isomerization of the pyridine ring has affected the configuration and conjugation of the molecules, resulting in different photophysical properties. From ***m*-DBCNPy** to ***o*-DBCNPy** and to ***p*****-DBCNPy**, the absorption and fluorescence emission peaks were bathochromically shifted in solutions and as neat films due to the enhanced intramolecular conjugation. Especially, ***o*-DBCNPy** exhibited the highest fluorescence quantum yield of 0.81 and the longest fluorescence lifetime of 7.96 ns as neat film of all three isomers. Moreover, non-doped organic OLEDs were fabricated using the molecules as the light-emitting layer. The best EL performance of all devices was achieved by the one based on ***o*-DBCNPy**. The maximum CE, PE, and EQE were 18.95 cd A^−1^, 15.76 lm W^−1^, and 4.31%, respectively, with color coordinates of (0.26, 0.70), which were close to that of the pure green color. The EL performances were mainly derived from the high fluorescence quantum yield of the ***o*-DBCNPy** neat film. Therefore, the delicate molecular tuning of AIEgens could provide them with enhanced photophysical properties, which makes them promising candidates for organic photoelectronic devices.

## Figures and Tables

**Table 1:** Photophysical data of the isomers in 1 × 10^−5^ M THF solutions.

Compound	*λ*_abs_ (nm)	*λ*_emi_ (nm)	Stokes Shift (nm)	*τ*_f_ (ns)	*Φ*_f,s_ *^a^*	*k*_f_ *^b^* (s^−1^)	*k*_nr_ *^b^* (s^−1^)
***o*-DBCNPy**	408	549	141	1.10	0.11	1.00 × 10^8^	8.09 × 10^8^
***m*-DBCNPy**	402	541	139	0.85	0.10	1.18 × 10^8^	1.06 × 10^9^
***p*-DBCNPy**	413	566	153	0.81	0.08	9.88 × 10^7^	1.14 × 10^9^

*^a^* Fluorescence quantum yield in THF solutions. Rhodamine B (0.7 in ethanol) was used as the reference [18]. *^b^ k*_f_, radiative decay rate constant (*k*_f_ = *Φ*_f,s_/*τ*_f_); *k*_nr_, nonradiative decay rate constant (*k*_nr_ = 1/*τ*_f_ − *k*_f_) [43].

**Table 2:** Fluorescence properties of the neat films of the molecules.

Compound	*λ*_emi_ (nm)	*τ*_f_ *^a^* (ns)	*Φ*_f,f_ *^a^*	*k*_f_ *^b^* (s^−1^)	*k*_nr_ *^b^* (s^−1^)
***o*-DBCNPy**	543	7.96	0.81	1.02 × 10^8^	2.36 × 10^7^
***m*-DBCNPy**	541	3.32	0.47	1.42 × 10^8^	1.60 × 10^8^
***p*-DBCNPy**	549	5.94	0.57	9.60 × 10^7^	7.24 × 10^7^

*^a^* Absolute fluorescence quantum yields of the films (*Φ*_f,f_) were obtained by a calibrated integrating sphere. *^b^ k*_f_, radiative decay rate constant (*k*_f_ = *Φ*_f,s_/*τ*_f_); *k*_nr_, nonradiative decay rate constant (*k*_nr_ = 1/*τ*_f_ − *k*_f_).

**Table 3:** Electrochemical data of the isomers.

Compound	*E*_ox_ *^a^* (V)	*E*_red_ *^a^* (V)	IP *^b^* (eV)	EA *^c^* (eV)
***o*-DBCNPy**	0.60	−1.98	5.53	2.95
***m*-DBCNPy**	0.62	−2.01	5.55	2.92
***p*-DBCNPy**	0.61	−1.93	5.54	3.00

*^a^* Oxidation (reduction) potential, estimated from the onset of the first oxidation (reduction) wave, V versus Ag/Ag^+^; *^b^* vertical ionization potential, IP = 4.93 eV + *E*_ox_ (the value (minus) 4.93 eV was obtained from the calibration of the Ag^+^ electrode versus vacuum) [44]; *^c^* vertical electron affinity, EA = 4.93 eV + *E*_red_.

**Table 4:** Electroluminescence (EL) performance of non-doped OLEDs based on the molecules.

Devices	Maximum Brightness (cd m^−2^)	Maximum Current Efficiency (CE) (cd A^−1^)	Maximum Power Efficiency (PE) (lm W^−1^)	Maximum External Quantum Efficiency (EQE) (%)	CIE Coordinates
***o*-DBCNPy**	4487	18.95	15.76	4.31	0.26, 0.70
***m*-DBCNPy**	2561	14.32	11.54	3.25	0.25, 0.71
***p*-DBCNPy**	3604	16.88	14.59	3.99	0.29, 0.67

## Data Availability

The data presented in this study are available in the Supplementary Materials.

## Supplementary Materials

The following supporting information can be downloaded at https://www.mdpi.com/article/10.3390/molecules28073282/s1. Figure S1: ^1^H NMR (400 MHz, CDCl_3_) spectrum of compound ***o*-DBCNPy**; Figure S2: ^13^C NMR (100 MHz, CDCl_3_) spectrum of compound ***o*-DBCNPy**; Figure S3: HRMS spectrum of compound ***o*-DBCNPy**; Figure S4: ^1^H NMR (400 MHz, CDCl_3_) spectrum of compound ***m*-DBCNPy**; Figure S5: ^13^C NMR (100 MHz, CDCl_3_) spectrum of compound ***m*-DBCNPy**; Figure S6: HRMS spectrum of compound ***m*-DBCNPy**; Figure S7: TGA curves of ***o*-DBCNPy**, ***m*-DBCNPy**, and ***p*-DBCNPy**; Figure S8: Absorption (a) and fluorescence emission (b) spectra of the non-doped film of the compounds; Figure S9: Absorption spectra of ***o*-DBCNPy** (a), ***m*-DBCNPy** (b), and ***p*-DBCNPy** (c) in THF/water mixtures. Concentration: 1 × 10^−5^ M; Figure S10: DSC curves of ***o*-DBCNPy** (a) and ***m*-DBCNPy** (b) in different states.

## Sample Availability

Samples of the compounds ***o*-DBCNPy**, ***m*-DBCNPy**, and ***p*****-DBCNPy** are available from the authors.
