# Supplementary material for: Tuning Photophysical Properties via Positional Isomerization of the Pyridine Ring in Donor–Acceptor-Structured Aggregation-Induced Emission Luminogens Based on Phenylmethylene Pyridineacetonitrile Derivatives
Source: Molecules. 2023 Apr 6;28(7):3282. doi: 10.3390/molecules28073282 (PMC10096500; doi:10.3390/molecules28073282)
Supplement: Supplementary file 1 [file molecules-28-03282-s001.zip › molecules-2314553-supplementary.pdf]

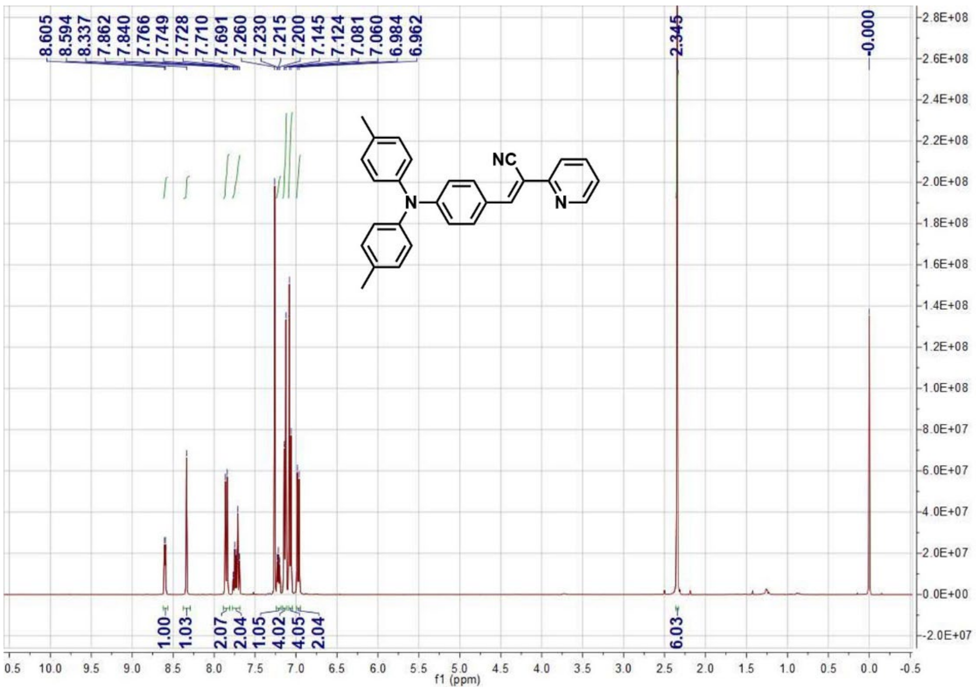

Figure S1. ^{1}H NMR (400 MHz, $CDCl_3$) spectrum of compound ***o*-DBCNPy**.

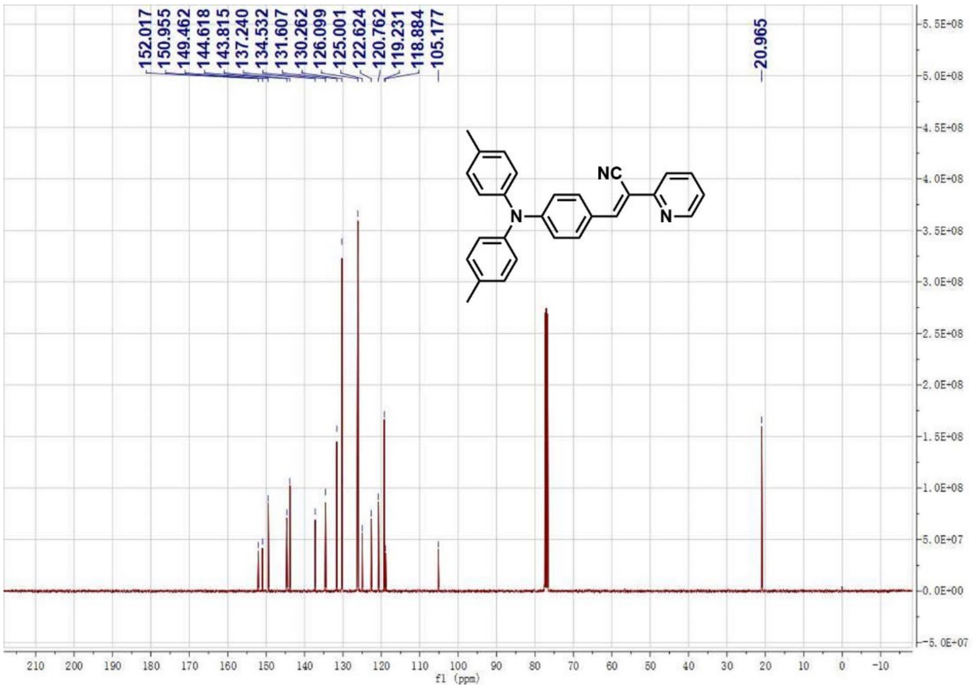

Figure S2. ^{13}C NMR (100 MHz, $CDCl_3$) spectrum of compound ***o*-DBCNPy**.

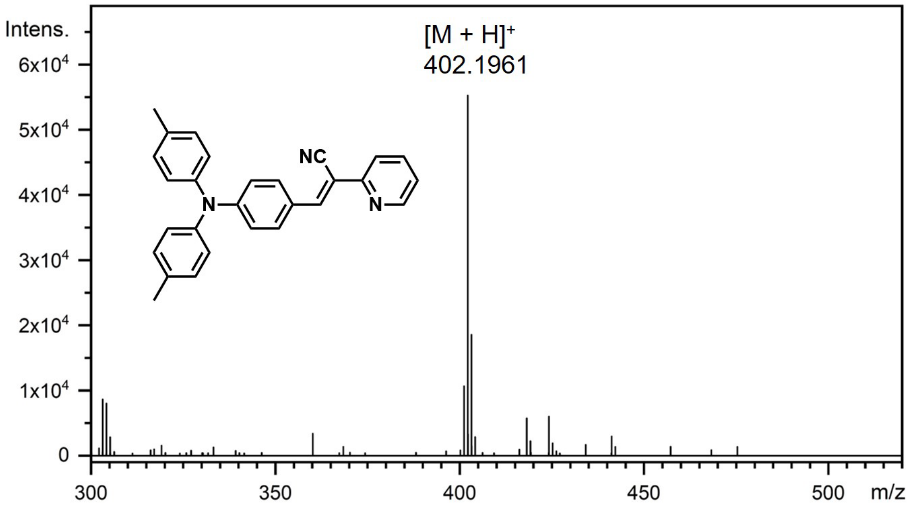

Figure S3. HR-MS spectrum of compound ***o*-DBCNPy**.

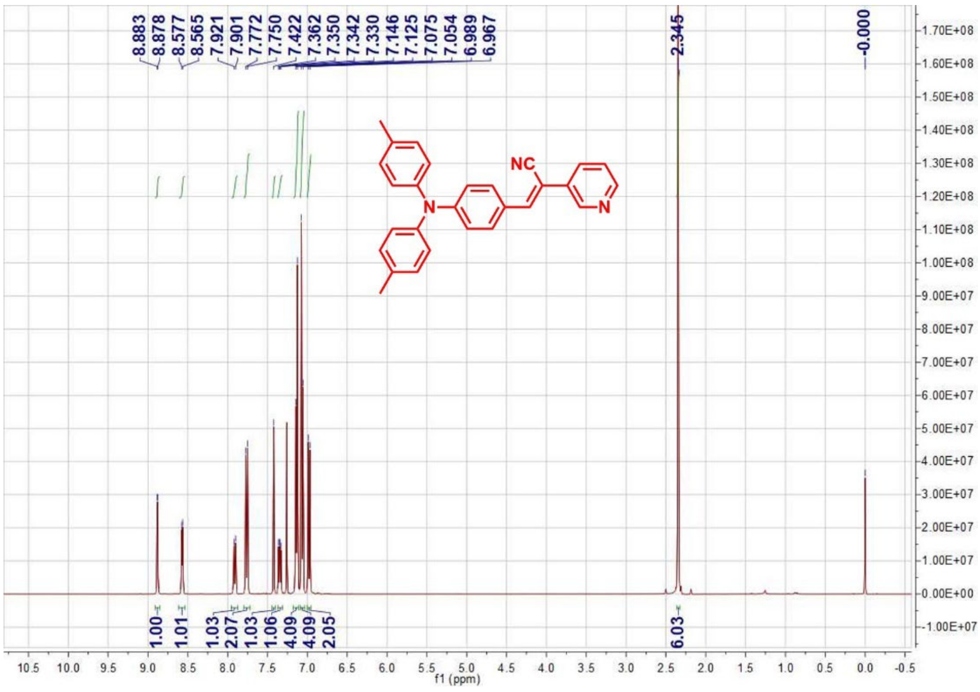

Figure S4. ^{1}H NMR (400 MHz, $CDCl_3$) spectrum of compound ***m*-DBCNPy**.

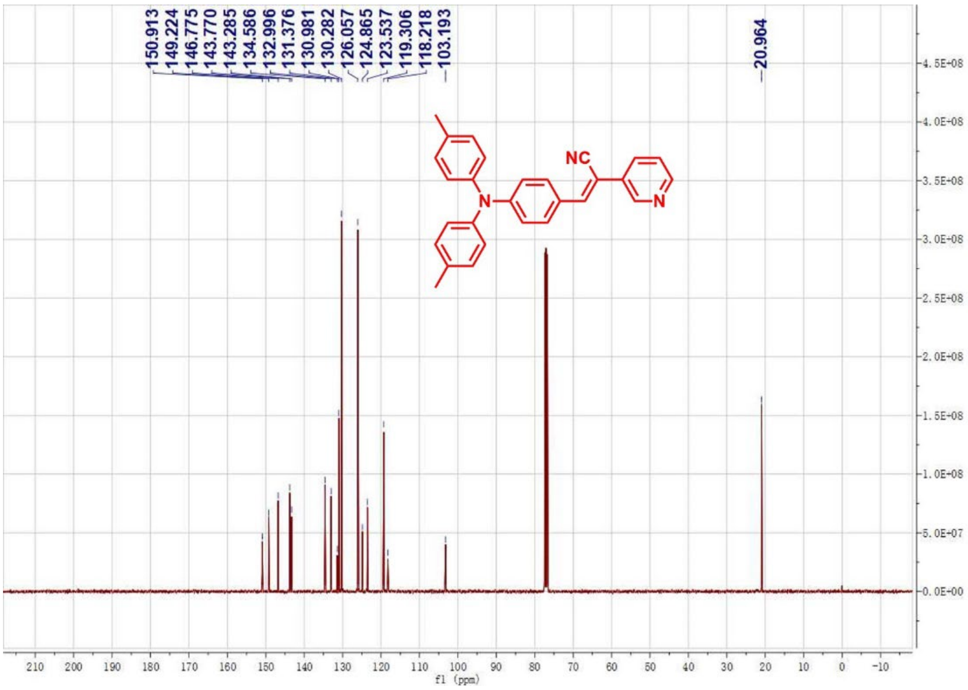

Figure S5. ^{13}C NMR (100 MHz, $CDCl_3$) spectrum of compound ***m*-DBCNPy**.

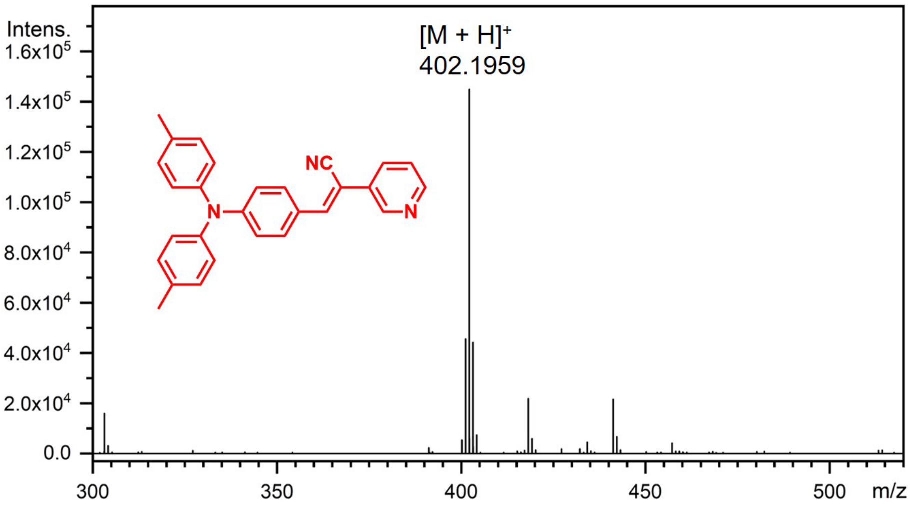

Figure S6. HR-MS spectrum of compound ***m*-DBCNPy**.

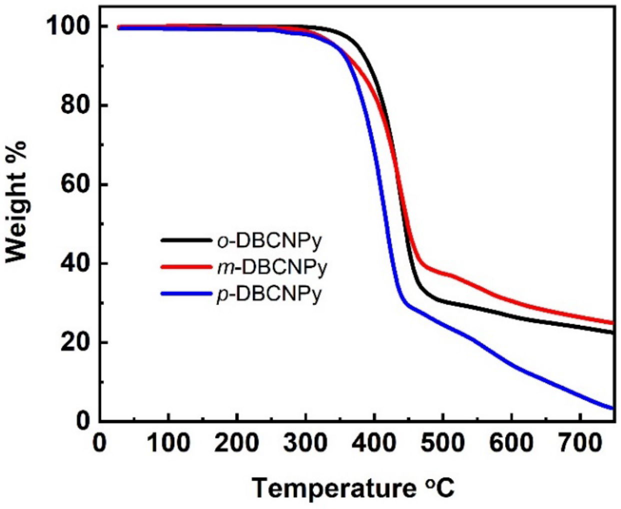

Figure S7. TGA curves of ***o*-DBCNPy**, ***m*-DBCNPy** and ***p*-DBCNPy**.

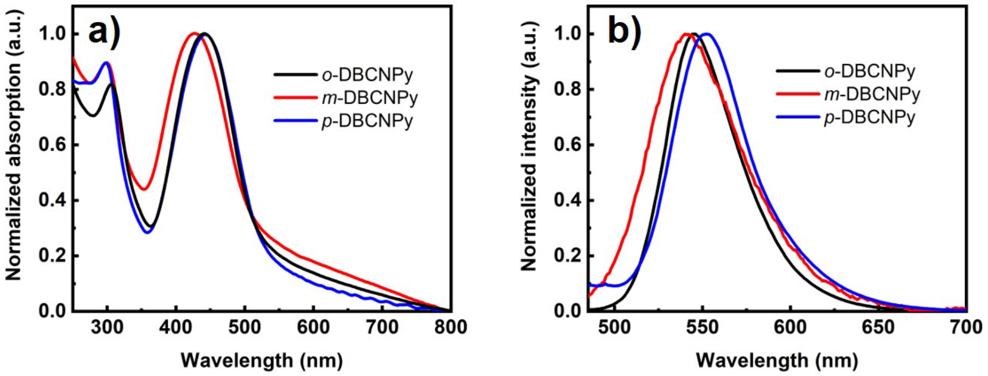

Figure S8. Absorption (a) and fluorescence emission (b) spectra of the non-doped film of the compounds.

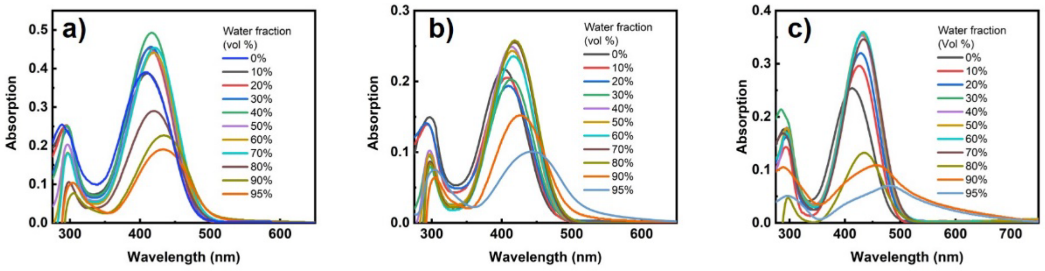

Figure S9. Absorption spectra of ***o*-DBCNPy** (a), ***m*-DBCNPy** (b) and ***p*-DBCNPy** (c) in THF/water mixtures. Concentration: 1×10^{-5} M.

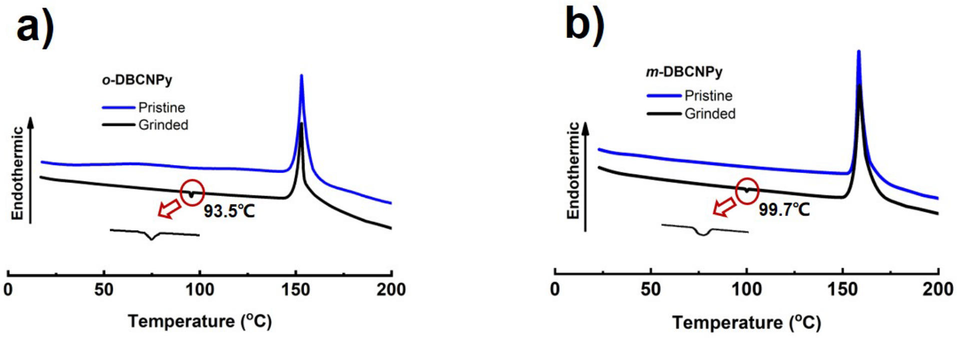

Figure S10. DSC curves of ***o*-DBCNPy** (a) and ***m*-DBCNPy** (b) in different states.
